# A water-soluble β-glucan improves growth performance by altering gut microbiome and health in weaned pigs

**DOI:** 10.1016/j.aninu.2021.04.006

**Published:** 2021-10-01

**Authors:** Yuliang Wu, Xue Li, Hongnan Liu, Yanjun Du, Jian Zhou, Lijun Zou, Xia Xiong, Huilin Huang, Zhiliang Tan, Yulong Yin

**Affiliations:** a Hunan Province Key Laboratory of Animal Nutritional Physiology and Metabolic Process, Key Laboratory of Agro-Ecological Processes in Subtropical Region, Institute of Subtropical Agriculture, National Engineering Laboratory for Pollution Control and Waste Utilization in Livestock and Poultry Production, Scientific Observing and Experimental Station of Animal Nutrition and Feed Science in South-Central, Institute of Subtropical Agriculture, Chinese Academy of Sciences, Changsha, 410125, Hunan, China; b Sichuan Synlight Biotech Ltd., Chengdu, 610041, China; c University of Chinese Academy of Sciences, Beijing, China; d Laboratory of Basic Biology, Hunan First Normal University, Changsha, 410205, China; e Hunan Co-Innovation Center for Utilization of Botanical Functional Ingredients, Changsha, 410128, China

**Keywords:** Beta-glucan, Small intestine, Weaned pig

## Abstract

Beta-glucan has been shown to have a beneficial effect on gastrointestinal health. This experiment was conducted to investigate the effects of β-glucan isolated from *Agrobacterium* sp. *ZX09* on growth performance and intestinal health of weaning pigs. A total of 108 weaned pigs (21 d of age; 6.05 ± 0.36 kg) were randomly divided into 3 groups (6 pens/group; 6 pigs/pen), and the groups were each treated with the following diets: 1) basal diet, 2) basal diet supplemented with 20 mg/kg olaquindox, 3) basal diet supplemented with 200 mg/kg β-glucan, for 21 d. Compared with the control group, pigs fed with 200 mg/kg β-glucan had greaterBW, average daily gain and duodenal villus height to crypt depth ratio (*P* < 0.05). Olaquindox increased the duodenal or jejunal villus height of pigs compared with β-glucan. Compared with the control group, β-glucan tended to increase the occludin mRNA expression in the jejunum (0.05 < *P* < 0.10). Beta-glucan enriched the beneficial microbiota in the ileum of pigs (*P* < 0.05). In conclusion, β-glucan may promote growth performance by improving intestinal health and increasing beneficial microbiota of weaned pigs. The study results will provide valuable theoretical guidance for the utilization of β-glucan in weaned pigs.

## 1 Introduction

As one of the most stressful events that the piglets confront in swine production, weaning results in reduced feed intake and growth rate, and increased morbidity and mortality of weaned pigs (Pluske et al., 1997; Xiong et al., 2019). Intestinal health is critically important for the digestion and absorption of nutrients and the maintenance of the host barrier function against harmful pathogens and antigens. Weaning impairs the intestinal morphology, immune system, and barrier function of pigs (Hu et al., 2013). For decades, antibiotics have been recognized as growth promoters in animal production, however, many of them have been banned for use due to their resistance to pathogenic bacterial strains and residual contamination in animal production (Han KNK et al., 2007; Xiong et al., 2015; Zou et al., 2019). Therefore, substitutes for in-feed antibiotics have attracted the interests of researchers.

Beta-glucans, a linear polysaccharide of D-glucose monomers linked by β-1,3-glycosidic or β-1,4-glycosidic linkages, are abundant biopolymers and are found in the cell walls of cereals, bacteria, yeast, or fungi (Sener et al., 2007). Like dietary fibers, β-glucans affect host health in domestic animals or humans by selectively stimulating the intestinal bacteria growth or activity (Wood, 2007). Further, β-glucans have been shown to stimulate specific or nonspecific immune responses (Suzuki et al., 1990). However, the efficacy of β-glucans may be related to their functional properties, such as purity, structure and molecular weight. In the present study, a water-soluble β-glucan extracted from *Agrobacterium* sp. *ZX09* was investigated, which has a large and complex molecular structure and is mainly composed of ꞵ-1,3-D-glucan. Previous studies have showed that it could reduce obesity induced by a high-fat diet (Zhang et al., 2013), enrich beneficial flora and increase short-chain fatty acids content in the cecum of mice (Zhou et al., 2014b). It can also alleviate the inflammation of the colon induced by dextran sulfate sodium in mice (Zhou et al., 2014a). Moreover, another study showed that 100 mg/kg dosage of β-glucan from *Agrobacterium* sp. *ZX09* (purity ≥90%) could improve growth performance and intestinal function in weaned pigs (Luo et al., 2019b). Luo et al. also reported that it could improve growth performance, nutrient digestibility and pork quality of finishing pigs (Luo et al., 2019c). These results indicated that this water-soluble β-glucan might exert beneficial effects on animal gastrointestinal health.

However, whether it can be used as an in-feed substitute for antibiotics to improve intestinal health and growth performance in weaned pigs remains unknown. Therefore, in this study, we explored the effects of β-glucan as an in-feed substitute for antibiotics on the growth performance, intestinal morphology, immunity and antioxidant index, and microbiota community of weaned pigs.

## 2 Materials and methods

### 2.1 Animal ethics

The experimental design and procedures used in this study were approved by the Animal Care and Use Committee of the Institute of Subtropical Agriculture, Chinese Academy of Sciences (2013020).

### 2.2 Materials and reagent

Beta-glucan was extracted from the fermentation broth of *Agrobacterium* sp. *ZX09* (Sichuan Synlight Biotech Ltd, Chengdu, China). The average molecular weight of β-glucan was about 2,000 kDa and its purity was more than 60%. Olaquindox (purity ≥ 98%) was purchased from Shanghai yuanye Bio-Technology Co., Ltd.

### 2.3 Animals and experimental treatments

A total of 108 piglets (Duroc × [Landrace × Yorkshire]; 6.05 ± 0.36 kg) weaned at 21 d of age were used in this experiment. Pigs were blocked by sex and litter and were randomly assigned into 3 dietary treatments (6 pens/group; 6 pigs/pen) consisting of a basal diet (control group), the basal diet supplemented with 20 mg/kg olaquindox or 200 mg/kg β-glucan for 21 d. The basal diet was formulated to meet the nutrient requirements for weaned pigs (NRC, 2012), and the diet contained 54% corn (8.5% CP), 12% soybean meal (43% CP), 2.5% glucose, 2.5% whey powder, 5% fish meal, 6% puffed corn, 10% fermented soybean meal, 3% soybean oil, and 5% premix. All pigs were fed in an environmentally controlled nursery barn with a hard-plastic slatted floor and nipple drinkers. Pigs were allowed to drink and eat freely. The pigs were weighed and recorded at the beginning of the experiment and on the 14th and 21st day of the experiment.

### 2.4 Sample collection

On d 22, two pigs (1 female and 1 male) were randomly selected from each pen and euthanized by injection with an overdose of sodium pentobarbital solution (40 mg/kg BW) and then bled (Xiong et al., 2015). The liver and spleen were collected and weighed. Duodenum, mid-jejunum, and ileum were collected, rinsed several times with precooled saline and then divided into 2 parts. One part was used for collecting mucosa, the other part (approximately 1 to 2 cm) was fixed with 4% formaldehyde-phosphate buffer and kept at 4 °C for a microscopic assessment of the intestinal morphology. After the mucosa cell layers were scraped off, they were rapidly frozen in liquid N. Duodenum was collected from the junction of the stomach and the small intestine, jejunum was dissected in the middle of the small intestine, and ileum was collected anterior to the ileocecal junction.

### 2.5 Measurement of mucosal cytokines, intestinal antioxidant capacity and morphology

Intestinal mucosa was homogenized in precooled phosphate buffer solution, then the homogenate was centrifuged at 10,000 × *g* for 10 min at 4 °C and the supernatant was collected to measure the cytokine content and antioxidant capacity. The levels of interleukin (IL) -1β, IL-2, and IL-10 in the mucosa were measured using a commercially available enzyme-linked immunosorbent assay (ELISA) kit (Cusabio Biotech Co., Ltd., Hubei, China) according to the instructions provided by the manufacturer. Antioxidant capacity in jejunal mucosal supernatant was evaluated by determination of superoxide dismutase (SOD), malondialdehyde (MDA), total antioxidant capacity (T-AOC) and glutathione (GSH) with commercial kits (Nanjing Jiancheng Bioengineering Institute, Nanjing, China). The protein concentration in each sample was quantitated and standardized before the analysis of intestinal mucosal cytokines, and antioxidant capacity.

The intestinal segment approximately 1 cm in length was fixed in low melting paraffin, sliced into 5 μm thick sections, and dyed with hematoxylin and eosin solution. The villi height and crypt depth were measured using an optical microscope at a combined magnification of 40. Ten well-oriented intact villi and their associated crypts were detected per sample (Xiong et al., 2015).

### 2.6 Real-time PCR

Total RNA extraction of intestinal mucosa was performed using TRIZOL reagent (Invitrogen, Carlsbad, CA), and then the first-strand cDNA was synthesized using the cDNA synthesis kit (TaKaRa, Dalian, China). Real-time PCR was performed on QuantStudio 5 Real-Time PCR System (Applied Biosystems, Foster City, CA, United States) for quantitative analysis of occludin and zonula occludens-1 (*ZO-1*) mRNA with SYBR Green qPCR Master Mix (Thermo Fisher, Sunnyvale, CA, United States). All of the real-time PCR were run in triplicate, and the mRNA expressions, relative to *β-actin*, were calculated using the 2^−ΔΔCt^ method (Xiong et al., 2015). All primers were synthesized by TsingKe Biological Technology (Changsha, China) and their sequences are provided in Appendix Table 1.

### 2.7 Ileum DNA extraction and PCR amplification

The total bacterial DNA of ileal content (approximately 0.25 g) was extracted using a QIAamp DNA Stool Mini Kit (Qiagen, Hilden, Germany) according to the manufacturer's instructions. Bacterial community composition and diversity in each ileal sample were determined by high-throughput sequencing of microbial 16S rDNA genes. The V4 regions of 16S rRNA genes were amplified used a specific primer with the barcode (515F: 5′-GTGCCAGCMGCCGCGGTAA-3′ and 806R: 5′-GGACTACHVGGGTWTCTAAT-3′). Paired-end sequencing was processed on the IlluminaHiSeq2500 platform (Novogene, Beijing, China). The paired-end reads were distributed to the respective samples according to different marker sequences, and FLASH software was used to perform paired-end reads (Magoc and Salzberg, 2011). The effective tags were obtained by using the UCHIME algorithm (v7.0.1001) compared with the reference database (Edgar et al., 2011; Haas et al., 2011). Sequences were analyzed with Uparse software (v7.0.1001) and sequences with similarity greater than 97% were classified into the same operational taxonomic units (OTU) (Edgar, 2013). OTU were assigned to a taxonomy using the Ribosomal Database Project classifier program (v.2.20) (http://rdp.cme.msu.edu/). Alpha diversity analysis was applied to assess the complexity of species diversity and beta diversity analysis was used to assess the differences in species complexity of samples. Finally, functional metagenomes of the intestinal flora were predicted using PICRUSt based on the Greengenes 16S rRNA database (http://greengenes.secondgenome.com/) and Kyoto Encyclopedia of Genes and Genomes (KEGG) orthologs (Parks et al., 2014).

### 2.8 Statistical analysis

All data were verified for normal distribution by the UNIVARIATE procedure and analyzed by the PROC MIXED using SAS software (Version 9.2; SAS Institute Inc., Cary, NC, USA). The treatment was regarded as the fixed effect, and blocks and pigs were regarded as random effects in the statistical model. Least squares mean of treatment effect were calculated and the mean separation was performed through PDIFF function when significant treatment effect was detected. The statistical significance criterion was *P*-value < 0.05, whereas 0.05 ≤ *P*-value < 0.10 was considered to have trend towards significance.

## 3 Results

### 3.1 Growth performance, organ index, and intestinal morphology

On d 21, pigs fed with β-glucan had greater BW and average daily gain (ADG) than pigs in the control group (*P* < 0.05). Pigs fed with β-glucan tended to decrease the feed-to-gain (F:G) ratio compared with pigs in the control group (0.05 < *P* < 0.10; Table 1). Pigs fed with β-glucan significantly reduced crypt depth compared with the olaquindox group in the duodenum and jejunum (*P* < 0.05; Table 2). Pigs fed with laquindox had greater jejunal villus height-to-crypt depth (VH:CD) ratio than that in the control group (*P* < 0.05; Table 2).

**Table 1:** Effect of β-glucan on the growth performance and organ weight of weaned pigs^1^.

Item	Control	Olaquindox	β-glucan	SEM	*P-*value
IBW, kg	6.01	6.09	6.04	0.02	0.531
BW at 14 d, kg	7.90	7.99	8.15	0.07	0.487
BW at 21 d, kg	9.46^b^	9.80^ab^	10.09^a^	0.18	0.029
ADG from 1 to 14 d, g	135.17	143.91	151.19	4.63	0.541
ADG from 14 to 21 d, g	222.22^b^	256.13^ab^	276.79^a^	15.91	0.030
ADG from 1 to 21 d, g	164.19^b^	176.63^b^	193.06^a^	8.36	0.011
ADFI from 1 to 14 d, g	215.88	212.22	230.26	5.52	0.358
ADFI from 14 to 21 d, g	297.62^b^	307.95^ab^	311.02^a^	4.05	0.034
ADFI from 1 to 21 d, g	237.82	238.85	253.28	4.99	0.185
F:G from 1 to 14 d, g/g	1.62	1.50	1.52	0.04	0.365
F:G from 1 to 21 d, g/g	1.44	1.34	1.30	0.04	0.062
Organ weight, g					
Liver	279.06	273.23	306.17	7.53	0.161
Spleen	19.06	22.68	22.68	1.09	0.295

IBW = initial body weight; ADG = average daily weight gain, ADFI = average daily feed intake, F:G = feed:gain.

^a,b^ Within a row, values with different superscripts are significantly different (*P* < 0.05).

1 Six replicate pens of 6 pigs/pen for performance data.

**Table 2:** Effect of β-glucan on the intestinal morphology of weaned pigs^1^.

Item	Control	Olaquindox	β-glucan	SEM	*P*-value
Villus height, μm					
Duodenum	444.14^a^	500.93^a^	388.56^b^	12.94	<0.001
Jejunum	375.95^b^	496.76^a^	401.86^b^	14.53	0.002
Ileum	380.36^b^	487.83^a^	372.52^b^	14.66	<0.001
Crypt depth, μm					
Duodenum	233.43^a^	230.30^a^	170.82^b^	7.16	<0.001
Jejunum	200.33^ab^	220.23^a^	196.26^b^	4.15	0.026
Ileum	166.99^ab^	187.70^a^	160.43^b^	5.35	0.086
Villus height-to-crypt depth ratio, μm:μm					
Duodenum	2.03	2.19	2.29	0.07	0.256
Jejunum	1.88^b^	2.32^a^	2.06^ab^	0.07	0.036
Ileum	2.21	2.53	2.23	0.09	0.241

^a,b^ Within a row, values with different superscripts are significantly different (*P* < 0.05).

1 Six replicate pens of 2 pigs/pen for intestinal morphology data.

### 3.2 Antioxidant index, intestinal barrier function, and mucosal cytokine

There was no significant change on jejunal antioxidant index among the 3 groups (Table 3). Dietary supplementation with β-glucan tended to increase the mRNA expression of occludin on the jejunum compared with the other groups (0.05 < *P* < 0.10; Table 4). Compared with the control group, dietary supplementation with olaquindox tended to increase the IL-10 concentration on the ileum (0.05 < *P* < 0.10; Table 5).

**Table 3:** Effect of β-glucan on jejunum mucosal antioxidant capacity of weaned pigs^1^.

Item	Control	Olaquindox	β-glucan	SEM	*P*-value
MDA， nmol/mg protein	1.18	1.08	0.90	0.07	0.221
TSOD, U/mg protein	7.91	8.43	8.04	0.17	0.495
GSH, μmol/g protein	7.63	8.74	8.02	0.29	0.331
TAOC, U/mg protein	1.01	1.41	1.31	0.11	0.339

MDA = malondialdehyde; GSH = glutathione; TAOC = total antioxidant capacity; TSOD = total superoxide dismutase.

1 Six replicate pens of 2 pigs/pen for antioxidant capacity data.

**Table 4:** Effect of β-glucan on occludin and *ZO-*1 mRNA expression in intestinal mucosal of weaned pigs^1^.

Item	Control	Olaquindox	β-glucan	SEM	*P*-value
Duodenum					
Occludin	1.00	0.90	1.10	0.07	0.516
*ZO-1*	1.00	1.30	1.34	0.09	0.281
Jejunum					
Occludin	1.00	1.08	1.42	0.08	0.051
*ZO-1*	1.00	0.88	1.34	0.09	0.101
Ileum					
Occludin	1.00	1.58	0.71	0.18	0.118
*ZO-1*	1.00	1.22	0.75	0.12	0.326

*ZO-1 =* zonula occludens-1.

1 Six replicate pens of 2 pigs/pen for mRNA expression data.

**Table 5:** Effect of β-glucan on intestinal mucosal IL-1β, IL-2 and IL-10 of weaned pigs^1^.

Item	Control	Olaquindox	β-glucan	SEM	*P*-value
IL-1β, pg/g of protein					
Duodenum	3.20	7.28	5.33	1.17	0.116
Jejunum	4.59	5.03	4.47	0.70	0.809
Ileum	4.74	6.39	4.62	0.90	0.339
IL-2, pg/g of protein					
Duodenum	31.16	74.29	61.38	13.75	0.140
Jejunum	37.74	43.64	43.59	4.60	0.510
Ileum	46.93	62.11	48.65	12.70	0.667
IL-10, pg/g of protein					
Duodenum	7.51	12.98	12.38	1.64	0.115
Jejunum	9.85	10.07	9.85	1.22	0.983
Ileum	7.29^b^	16.16^a^	10.53^ab^	2.11	0.051

IL = interleukin.

^a,b^ Within a row, values with different superscripts are significantly different (*P* < 0.05).

1 Six replicate pens of 2 pigs/pen for enzyme-linked immunosorbent assay data.

### 3.3 Composition of the ileal microbiota of pigs

**Fig. 1:**
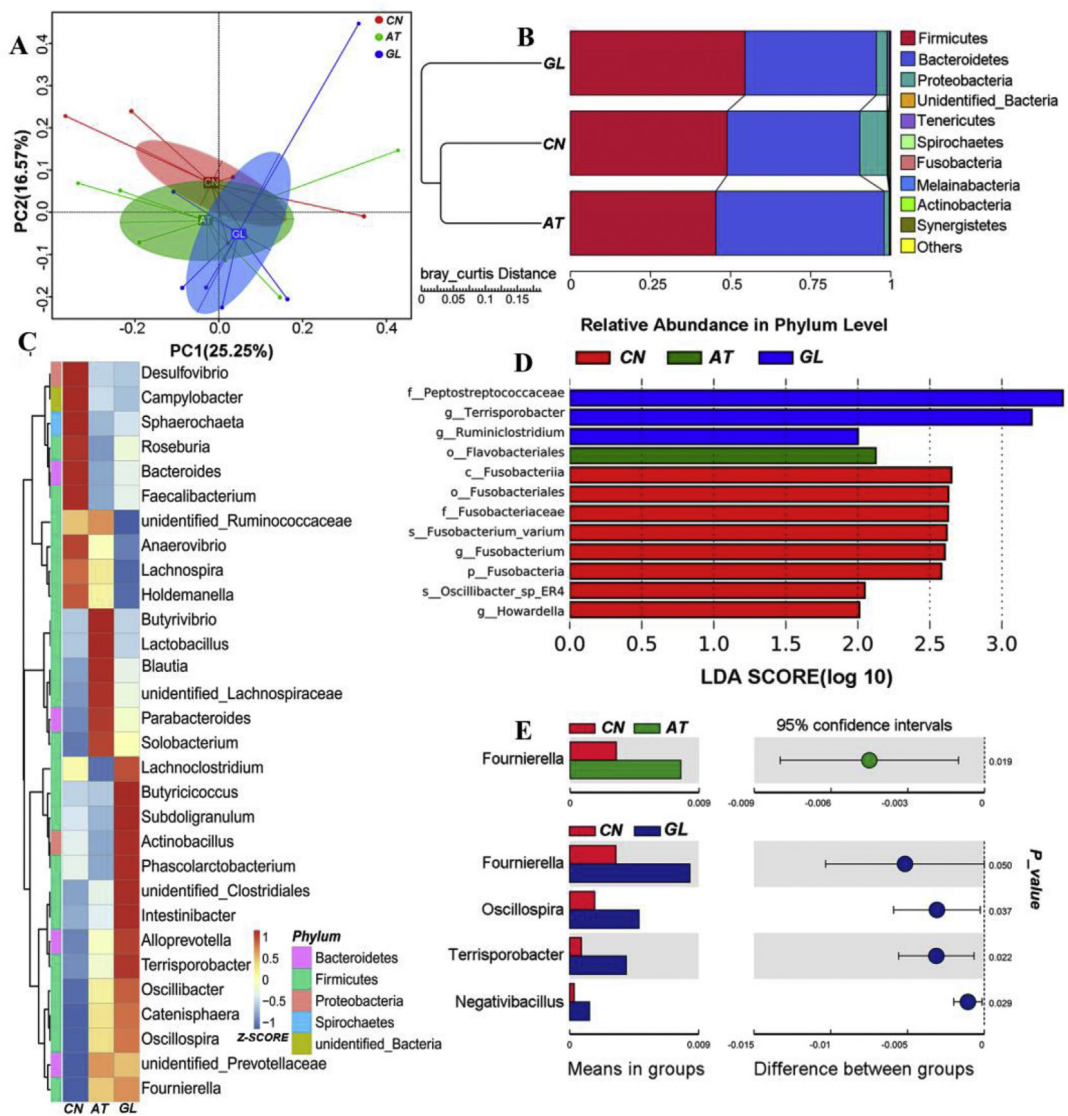
Alterations in ileal microbiota composition among 3 groups. **(A)** Principal coordinate analysis (PCoA) analysis based on the operational taxonomic units (OTU) table. **(B)** Unweighted Pair-group Method with Arithmetic Mean (UPGMA) analysis based on bray_curtis and OTU abundance at phylum level. **(C)** Heatmap tree shows the top 30 genera of all groups and their phylogenic relationships. The z-score indicates the richness and according to the Bray Curtis distance to cluster the genera in the clustering tree **(D)** LEfse analysis of different microorganism classification levels (linear discriminant analysis score = 2.0). **(E)** The *t*-test bar plot with significantly different genera between groups. CN: control group; AT: group with 20 mg/kg olaquindox; GL: group with 200 mg/kg β-glucan. PC = principal component.

The analysis showed that there were no significant differences in the sample diversity (Shannon, Simpson) and abundance indices (Chaol, ACE) among the 3 groups (Appendix Table 1). The principal coordinate analysis (PCoA) based on Bray–Curtis revealed that there were differences in microbiota among groups (Fig. 1A). *Firmicutes*, *Bacteroidetes* and *Proteobacteria* were dominant microbial divisions (Fig. 1B). The heatmap plot (according to the top 30, the most different genera) showed the relative abundance of genera in different groups (Fig. 1C). Linear discriminant analysis effect size (LEfSe) analysis of the ileal bacterial community showed that there were compositional differences between the β-glucan and control group (Fig. 1D). The relative abundance of *Fournierella*, *Oscillospira*, *Terrisporobacter* and *Negativibacillus* in the β-glucan group was higher than those in the control group (*P* < 0.05) and the relative abundance of *Fournierella* in the olaquindox group was higher than that in the control group (*P* < 0.05, Fig. 1E).

### 3.4 Changes in the metabolic functions of ileal microbiota

**Fig. 2:**
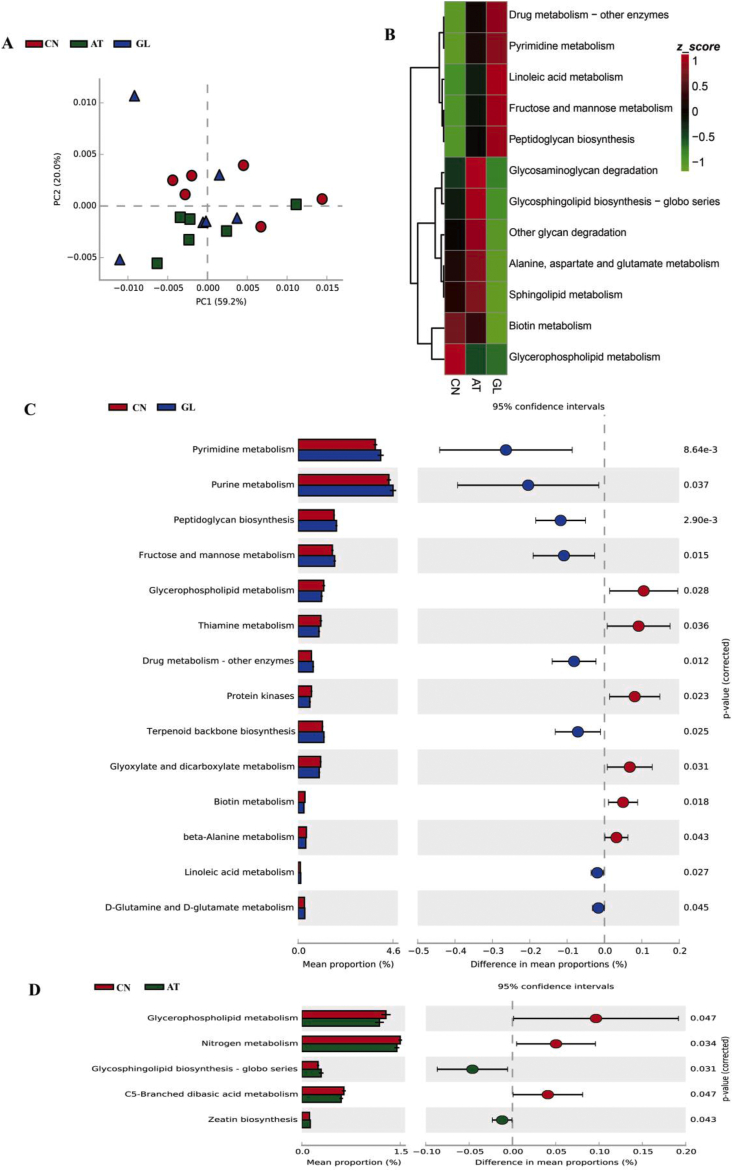
Functional differences in ileal microbiota among groups. **(A)** Principal components analysis (PCA) plot of functional profiles. **(B)** The functional profile significantly different among groups at the third level of Kyoto Encyclopedia of Genes and Genomes (KEGG) (metabolism related pathways only) and their phylogenic relationships was showed in the Heatmap tree. The z-score indicates the richness and according to the Bray Curtis distance to cluster the genera in the clustering tree. **(C)** The *t*-test bar plot with significantly different pathways between glucan group and control group at the KEGG level 3 **(D)** The *t*-test bar plot with significantly different pathways between antibiotic group and control group at the KEGG level 3. CN: control group; AT: group with 20 mg/kg olaquindox; GL: group with 200 mg/kg β-glucan. PC = principal component.

The principal component analysis based on KEGG annotation showed that the metabolic functions of ileal microbiota differed among groups. Twenty-two KEGG level 3 pathways that differed significantly among groups were observed via PICRUSt v1.1.3 (Appendix Table 2). Compared with the control group, pyrimidine metabolism, purine metabolism, peptidoglycan biosynthesis and fructose and mannose metabolism were increased in the β-glucan group (*P* < 0.05, Fig. 2B and C). Glycerophospholipid metabolism was decreased, and Zeatin biosynthesis was increased in the olaquindox group (*P* < 0.05, Fig. 2D).

## 4 Discussion

Weaning stress is generally accompanied by low growth, diarrhea and increased intestinal dysfunction. Olaquindox, as one of the quinoxaline-N, N-dioxides, was used as a therapeutic medicine to prevent bacterial enteritis in weaned pigs. Beta-glucans play an important role in modulating immunity function and promoting beneficial intestinal microflora growth (Suzuki et al., 1990; Wood, 2007). The weaning period is a critical phase for the functional differentiation of the immunity in pigs. In a previous study, weaned pigs supplemented with β-glucan had higher BW and ADG compared with pigs supplemented with a basal diet, and tended to decrease the F:G after 21 d feeding period. There was higher growth performance in weaned pigs supplemented with 200 mg/kg β-glucan than those with 100 mg/kg (data not shown), and we chose the weaned pigs supplemented with 200 mg/kg β-glucan for further analysis. In addition, we did not find any significant differences in growth performance after 21 d feeding period between the olaquindox and the control group, however, pigs fed with β-glucan had greater BW and ADG than pigs in other groups. These results showed that β-glucan could improve the growth rate of weaned pigs, which was consistent with the previous study on LPS-challenged pigs (Luo et al., 2019a). However, no significant difference was found on growth performance between the olaquindox and the control group; the reason may be attributed to the feeding time (21 d) or dosage of the olaquindox in the present study.

The principal function of the intestine focuses on the absorption of nutrients generated from intestinal digestion. Weaning stress usually accompanies intestinal atrophy, which further limits the digestive and absorptive capacity of the intestine in weaned pigs (Xiong et al., 2019). The villus height and VH:CD ratio are useful parameters for estimating the nutrient digestion and absorption capacity of the small intestine (Montagne et al., 2003). In the present study, compared with the control group, olaquindox significantly increased the small intestinal villus height and jejunal VH:CD ratio. As we know, bacterial species in the intestine compete with the host for nutrients. Although olaquindox did not significantly improve the growth performance of weaned pigs, it did increase the nutrient absorption capacity in the present study for its antibacterial capacity. These results indicate that the improvement of the growth rate of β-glucan on weaned pigs might be due to the increase in the digestive and absorptive capacity of the small intestine.

In addition to intestinal morphology analysis, intestinal antioxidant capacity and tight junction were also used to evaluate the intestinal health of weaned pigs. The epithelial barrier consists of an intact epithelial monolayer and the intercellular junctions (Moeser and Blikslager, 2007). Under oxidative stress conditions, the overproduction of reactive oxygen species may cause oxidative damage, impair the function of immune cells and cause the spread of pathogenic microorganisms, which could subsequently result in defects in the gastrointestinal barrier function (Gangwar et al., 2017). Compared with the control group, β-glucan tended to increase the mRNA expression of occludin on the jejunum. This result may suggest that β-glucan tended to improve the jejunal health of weaned pigs.

Immune stress can impact animal growth performance. Cytokines play a crucial role in the balance of the immune and inflammatory responses, which can regulate innate and adaptive immune responses to antigens and infectious agents. Pro-inflammatory cytokines mediate the host inflammatory response to prevent susceptibility to infection, and anti-inflammatory cytokines protect against intestinal inflammation (Praveena et al., 2010; Xiong et al., 2015). Interleukin-10 plays a key role in the tolerance to self- and mucosal antigens and can regulate intestinal endocrine activity (Sanjabi et al., 2009; Latorre et al., 2013). In the present study, olaquindox dietary supplementation up-regulated the expression of IL-10 in the ileum of pigs. However, the present study showed that β-glucan supplementation did not affect the secretion of intestinal cytokines. Further research is needed on the role of β-glucan on the intestinal immune system of pigs.

Beta-glucans, like fermentable dietary fibers, can be hydrolyzed in the large intestine by various enzymes and exist as poly- and oligomers. Like other β-glucans, β-glucan extracted from *Agrobacterium* sp. *ZX09* is not digested and absorbed by the small intestine but can be fermented by the hindgut flora. Zhou et al. showed that dietary β-glucan affected microbial communities and increased short-chain fatty acid levels in the cecum of mice (Zhou et al., 2014b). As we known, pigs have relatively low levels (103 to 105 cfu/g of digesta) of bacteria in the proximal gastrointestinal tract (stomach and duodenum), and the flora in the ileum is far more diverse (108 to 109 cfu/g) (Moughan et al., 1992). The present results showed that there were no significant differences in the diversity and richness of ileal microflora among the 3 groups. However, at the genus level, the relative abundance of *Fournierella*, *Oscillospira*, *Terrisporobacter* and *Negativibacillus* in the β-glucan group and *Fournierella* in the olaquindox group were significantly increased. Beta-glucan contains α-(1,3)-D-glycosidic bond structure, which may provide growth substrates for some strains with α-glucosidase activity, including *Fournierella* (Togo et al., 2017), *Parabacteria* and *Alistipes* (Zhou et al., 2014b). *Oscillospira* species is a butyrate producer, and previous studies have shown that it can reduce intestinal inflammatory diseases (Gophna et al., 2017). Moreover, the higher relative abundance of fructose, and mannose metabolism in the pigs supplemented with β-glucan may indicate that β-glucan could affect the carbohydrate metabolism of the body. Zhang et al. showed that β-glucan enhances lipid and glucose metabolism of mice (Zhang et al., 2013). These results may indicate that β-glucan can be fermented and metabolized to produce volatile fatty acids, therefore exerting a prebiotic promotion of beneficial bacteria.

## 5 Conclusion

In conclusion, 200 mg/kg β-glucan or 20 mg/kg olaquindox can improve the growth performance of weaned pigs. Beta-glucan may improve intestinal health by increasing the ileal beneficial microbiota of weaned pigs.

## Author contributions

**Yuliang Wu**, **Xue Li** and **Xia Xiong** designed the experiments; **Hongnan Liu**, **Xia Xiong** and **Yuliang Wu** conducted the experiments; **Jian Zhou** and **Lijun Zou** helped with animal experiments; **Yuliang Wu**, **Xue Li** analyzed the data; **Yuliang Wu** wrote the manuscript; **Xia Xiong** and **Yulong Yin** revised the manuscript. All authors read and approved the final manuscript.

## Conflict of interest

We declare that we have no financial and personal relationships with other people or organizations that can inappropriately influence our work, and there is no professional or other personal interest of any nature or kind in any product, service and/or company that could be construed as influencing the content of this paper.
