# Supplementary material for: A water-soluble β-glucan improves growth performance by altering gut microbiome and health in weaned pigs
Source: Anim Nutr. 2021 Oct 1;7(4):1345–51. doi: 10.1016/j.aninu.2021.04.006 (PMC8571503; doi:10.1016/j.aninu.2021.04.006)
Supplement: Supplementary file 1 [file mmc1.docx]

**Appendix**

**Table 1**

Primer pairs used for quantitative real-time PCR

| Gene | Primer sequence | Product size, bp | GenBank accession number |
| --- | --- | --- | --- |
| Occludin | F: 5'-ACCCAGCAACGACATA-3' | 155 | XM_005672525.3 |
|  | R: 3'-TCACGATAACGAGCATA-5' |  |  |
| *ZO-1* | F:5'-CCTGAGTTTGATAGTGGCGTTGA-3' | 269 | XM_021098896.1 |
|  | R: 3'-AAATAGATTTCCTGCTCAATTCC-5' |  |  |
| β-actin | F: 5'-GGATGCAGAAGGAGATCACG-3' | 130 | XM_021086047.1 |
|  | R: 3'-ATCTGCTGGAAGGTGGACAG-5' |  |  |

*ZO-1 =* zonula occludens-1.

**Table 2**

Changes in alpha diversity indices of ileal microbiota on weaned pigs (*n*= 6)

| Indices | Control | Antibiotic | β-glucan | SEM | *P*-value |
| --- | --- | --- | --- | --- | --- |
| Shannon | 5.938 | 5.767 | 5.887 | 0.407 | 0.957 |
| Simpson | 0.946 | 0.940 | 0.926 | 0.024 | 0.872 |
| Chao1 | 629.9 | 574.9 | 596.0 | 41.023 | 0.688 |
| ACE | 607.0 | 572.9 | 594.3 | 28.587 | 0.712 |

ACE=abundance-based coverage estimator.

**Table 3**

Significantly altered metabolic profiles among groups at Kyoto Encyclopedia of Genes and Genomes (KEGG) level 3.

| Observation Ids | | Effect size | | Control, % | | Antibiotic, % | | β-glucan, % | | | *P*-value | | |
| --- | --- | --- | --- | --- | --- | --- | --- | --- | --- | --- | --- | --- | --- |
| Alanine, aspartate and glutamate metabolism | | 0.404 | | 1.076 | | 1.107 | | 1.067 | | | 0.021 | | |
| Biotin metabolism | | 0.362 | | 0.161 | | 0.159 | | 0.138 | | | 0.034 | | |
| DNA replication proteins | | 0.379 | | 1.235 | | 1.313 | | 1.345 | | | 0.028 | | |
| Drug metabolism - other enzymes | | 0.357 | | 0.316 | | 0.346 | | 0.358 | | | 0.036 | | |
| Function unknown | | 0.415 | | 1.227 | | 1.156 | | 1.143 | | | 0.018 | | |
| Glycerophospholipid metabolism | | 0.390 | | 0.606 | | 0.570 | | 0.558 | | | 0.025 | | |
| Glycosaminoglycan degradation | | 0.341 | | 0.056 | | 0.080 | | 0.050 | | | 0.044 | | |
| Glycosphingolipid biosynthesis-globo series | | 0.463 | | 0.116 | | 0.142 | | 0.104 | | | 0.009 | | |
| Lysosome | | 0.407 | | 0.090 | | 0.121 | | 0.076 | | | 0.020 | | |
| Mismatch repair | | 0.427 | | 0.830 | | 0.872 | | 0.876 | | | 0.015 | | |
| NOD-like receptor signaling pathway | | 0.337 | | 0.047 | | 0.051 | | 0.053 | | | 0.046 | | |
| Nucleotide excision repair | | 0.330 | | 0.402 | | 0.426 | | 0.419 | | | 0.050 | | |
| Other glycan degradation | | 0.463 | | 0.292 | | 0.341 | | 0.249 | | | 0.009 | | |
| Other transporters | | 0.381 | | 0.258 | | 0.243 | | 0.264 | | | 0.027 | | |
| Peptidoglycan biosynthesis | | 0.443 | | 0.850 | | 0.895 | | 0.912 | | | 0.012 | | |
| Protein kinases | | 0.339 | | 0.316 | | 0.281 | | 0.278 | | | 0.045 | | |
| Pyrimidine metabolism | | 0.337 | | 1.826 | | 1.946 | | 1.966 | | | 0.046 | | |
| Replication, recombination and repair proteins | | 0.353 | | 0.766 | | 0.738 | | 0.825 | | | 0.038 | | |
| Secretion system | | 0.453 | | 1.354 | | 1.187 | | 1.227 | | | 0.011 | | |
| Sphingolipid metabolism | | 0.381 | | 0.235 | | 0.253 | | 0.205 | | | 0.027 | | |
| Thiamine metabolism | | 0.339 | | 0.536 | | 0.519 | | 0.495 | | 0.045 | | |  |
| Transcription machinery | | 0.448 | | 0.999 | | 1.036 | | 1.081 | | 0.012 | | |  |
